# Hydrogen (H_2_)/Toluene (TOL) Separation via One and Two Stages of the Bis(triethoxysily)ethane (BTESE) Membranes

**DOI:** 10.3390/membranes14080165

**Published:** 2024-07-25

**Authors:** Suhaina Mohd Ibrahim, Xin Yu, Shigeru Miyata, Kengo Mishina, Feridoun Salak, Sulaiman Oladipo Lawal, Toshinori Tsuru, Ken-ichi Sawamura

**Affiliations:** 1 eSep Inc., Keihanna Open Innovation Center@Kyoto (KICK), Annex 320, 7-5-1, Seikadai, Seika-cho, Soraku-gun, Kyoto 619-0238, Japansulaiman@esep.co.jp (S.O.L.); 2 Department of Chemical Engineering, Hiroshima University, 1-4-1 Kagamiyama, Higashihiroshima 739-8527, Japan

**Keywords:** hydrogen purification, hydrogen (H_2_)/toluene (TOL) gas mixtures, bis(triethoxysilyl)ethane (BTESE) membranes

## Abstract

The separation ability of bis(triethoxysilyl)ethane (BTESE) membranes for hydrogen (H_2_) purification from hydrogen (H_2_)/toluene (TOL) gas mixtures after a methylcyclohexane (MCH) dehydrogenation process was investigated via one-stage and two-stage membrane processes. This study revealed that BTESE membranes of varied pore sizes (0.4, 0.5, and 0.7 nm) in a one-stage configuration can manage to achieve a H_2_ purity ~99.9%. However, the TOL concentrations fell within a wide range, ranging from 280 to 5441 ppm. A primary goal of this research was to lower the TOL concentration in the permeate stream below 200 ppm. Hence, by applying the two-stage membrane, it was demonstrated that the TOL concentration in the permeate stream could be lowered below 200 ppm.

## 1. Introduction

Hydrogen (H_2_) is one of the potential renewable energy sources of the future [1,2,3,4,5]. The International Energy Agency (IEA) predicts that the world’s need for H_2_ will increase seven-fold to 520 Mt by 2070 [6]. Various types of inorganic membranes, such as metal, molecular sieving carbon, zeolites, and ceramic membranes [7,8,9], have been considered for H_2_ separation. As one of the new classes of membranes, organosilica membranes, especially BTESE-derived membranes, have been extensively investigated for the last decade since they show excellent H_2_ permeance (H_2_ permeance > 10^−6^ mol m^−2^ s^−1^ Pa^−1^ and H_2_/TOL > 1000) [10,11]. In the present study, the pore size distribution of our BTESE membranes was controlled by altering the number of coating layers of the BTESE solution [12,13] instead of focusing on different water ratios [14,15,16], firing temperature [17,18], doping with ZIF-7 [19], and copolymerization [20]. Previously, our membrane was successfully used in the separation of DMF and H_2_O [13]. Hence, we believe our in-house membranes are capable of H_2_/TOL separation as well.

Since H_2_ cannot be obtained directly from nature, it is a secondary energy source. Green H_2_ is produced by water electrolysis using electricity generated from solar or wind energy, whereas blue hydrogen is extracted alongside the CO_2_ emitted from the hydrocarbons in fossil fuels [21,22,23]. For both types of H_2_, the greatest difficulties in expanding the use of H_2_ energy are safe storage and transportation. To overcome this barrier, organic chemical hydrides such as methylcyclohexane (MCH) are proposed as hydrogen carriers because of their ease of handling.

It is well known that MCH, which catalytically decomposes to H_2_ and TOL, liquefies at room temperature and has a volume equivalent to several hundred thousandths of H_2_. Since MCH is stable at normal temperatures, it can be utilized in the existing fuel supply chain, including gas stations. Hence, H_2_ and TOL need to be separated at the use site. This is achieved by vaporizing the MCH and catalytic decomposition at 250–300 °C, followed by purification or separation of H_2_ and TOL using a ceramic membrane [14,24,25,26,27,28,29,30]. To achieve a high purity of H_2_, a typical process includes the condensation of decomposed MCH to separate gas (H_2_) and liquid product (TOL) from the MCH dehydrogenation reaction, followed by the purification of H_2_ gas from that containing saturated TOL vapor.

However, most of the researchers are focusing on the BTESE and silica membrane reactors coupled with catalysts [14,25,26,27,28] for the dehydrogenation of methylcyclohexane (MCH) to toluene (TOL). Only limited studies have been reported focusing on the production of high-purity H_2_ from the H_2_/TOL mixture. In the present study, we used H_2_ and TOL as our feed components instead of MCH. It is well known that H_2_ separation membranes can be used in two ways. The first approach involves a membrane for a one-stage purification process after the MCH dehydrogenation reaction. However, a one-stage membrane of extremely high selectivity is required to achieve the minimal specification of fuel cell application. So as another approach, a two-stage membrane is proposed for further purification. As the main goal of this study is to achieve H_2_ purity > 99.9% and a TOL concentration in the permeate stream below 200 ppm, the present study will only focus on the one-stage and two-stage BTESE membrane systems by using our own in-house-produced BTESE membrane.

## 2. Materials and Methods

### 2.1. Preparation of BTESE-Derived Sols and Membranes

The organosilica precursor BTESE was dissolved in an ethanol (EtOH) solution. After that, under vigorous agitation, dropwise additions of nitric acid (HNO_3_) and H_2_O were made with the final molar ratios of BTESE/H_2_O/HNO_3_ = 1/240/0.2. The concentration of BTESE was maintained at 5 weight percent (wt.%) by adjusting the amount of EtOH added to the solutions. After 6 h of continuous stirring at 25 °C, the solution was stored in a closed system to allow BTESE-derived organosilica sols to develop. It was then aged for 8 days at 50 °C before being used as a top layer.

Porous α-alumina tubes with an outer diameter of 12 mm and 400 mm in length, 50% porosity, and an average pore size of 3 µm were used as a support. The porous support was supplied by IWAO JIKI KYOGYO Co., Ltd., Saga Prefecture, Japan [12]. The exterior of the porous support was first covered with α-alumina particles, which had an average diameter of 0.2 µm. After that, the surface was fired for ten minutes at 550 °C to produce an α-alumina particle layer. Ultimately, the BTESE solution was coated, dried, and fired for 30 min at 300 °C under air to form the BTESE-derived organosilica top layer. Our previous manuscripts [12,13] provide a comprehensive overview of the membrane fabrication procedure.

**Figure 1:**
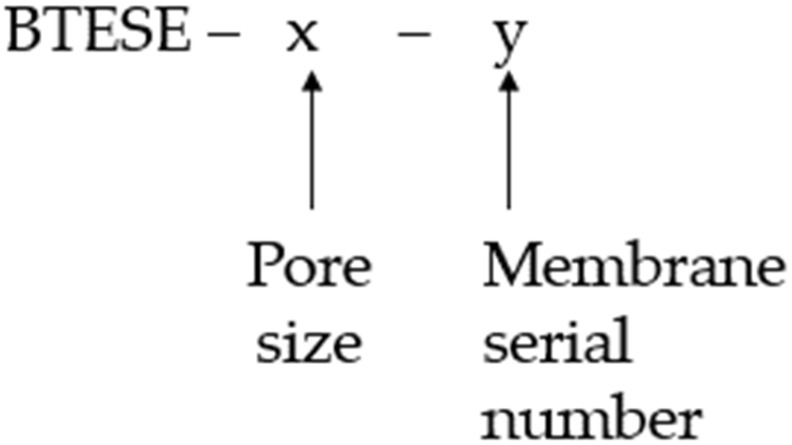
BTESE sample coding.

**Figure 2:**
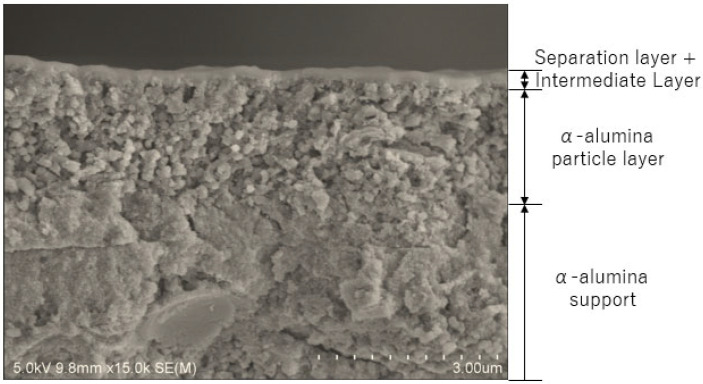
SEM image of a cross-section of a BTESE membrane.

In the present study, BTESE membranes are referred to as BTESE-x-y. The x and y letters indicate the membrane pore size and the membrane serial number, respectively, as shown in Figure 1. In the present study, there is 1 membrane with a pore size around 0.7 nm and another with a pore size around 0.5 nm and 4 membranes with a pore size around 0.4 nm, and Table 1 lists all the membranes that were used in this study. The morphologies of the BTESE membranes were examined using a Hitachi S-48000 field emission scanning electron microscope (FESEM)Hitachi High-Tech, Tokyo, Japan. Figure 2 shows a SEM image of a cross-section of an in-house-prepared BTESE membrane. This clearly shows that a crack-free continuous separation layer thickness below 3 μm was formed on the top of the intermediate and alumina layers after the calcination process at 300 °C. Other characterizations can be found elsewhere [11,14,16].

### 2.2. Single-Gas Permeation (SGP)

The SGP measurement was carried out at 200 °C using single components of He, H_2_, N_2_, CH_4_, CF_4_, and SF_6_. The permeate stream was kept at atmospheric pressure while the membrane module experienced a transmembrane pressure of 0.04–0.1 MPa. The gas permeance, *P* (mol m^−2^ s^−1^ Pa^−1^), is obtained from Equation (1). The gas flowrates of the permeate streams were monitored using a mass flow meter (Kofloc EX-700R model, Kyoto, Japan) with flow rates up to 100 mL/min and 5 L/min, while the pressure of the retentate stream was measured by a digital pressure manometer (DMS-7A, 200 kPa, Hitachi High-Tech, Tokyo, Japan).

(1) P=n/S∆p

Equation (1) uses *n*, or the permeate flow rate (mol s^−1^), as its unit. S represents the membrane surface area (m^2^) and Δ*p* stands for the transmembrane pressure differential (Pa). The ratio of gas permeance for A and B is defined as α_A_/_B_ (-) in Equation (2), which represents the optimal selectivity for A over B.

(2) $$\alpha_{A/B\;}=P_{A}/P_{B}$$

### 2.3. H_2_/TOL Separation Test

**Figure 3:**
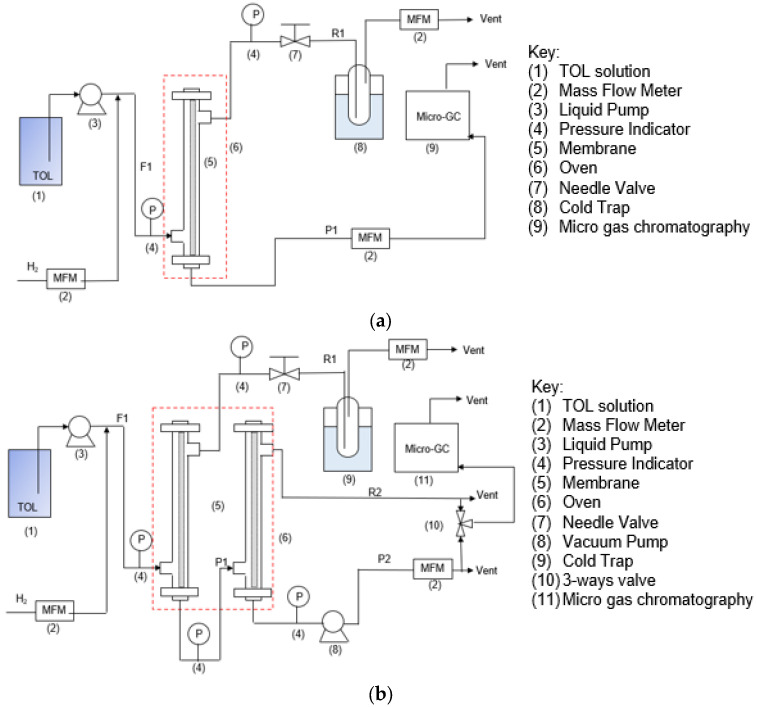
Hydrogen (H_2_)/toluene (TOL) separation, (**a**) one-stage and (**b**) two-stage membrane system configurations.

Figure 3a displays a schematic diagram of the one-stage gas separation device. The pressure of the feed (F1) stream was set at 200 kPaA and controlled using a needle valve. The pressure of the feed (F1) and retentate (R1) streams were measured by a digital pressure manometer (DMS-7A, 200 kPa, Hitachi High-Tech, Tokyo, Japan). The permeate (P1) stream was kept at atmospheric pressure. H_2_ was supplied directly from a gas cylinder at a flowrate of 4.94 L/min, while TOL was fed using a pump (LC-10 AD VP Shimadzu, Liquid Chromatography, Tokyo, Japan) at a flowrate of 1.2 mL/min to control the feed concentration at 30,000 ppm. The H_2_/TOL binary mixture was fed on the shell side of the membrane module, whose temperature was maintained at 200 °C. The gas flow rates of the feed (F1), retentate (R1), and permeate (P1) streams were monitored using a mass flow meter (Horiba, STEC, Z500 model). The Agilent 990 micro gas chromatography (Channel 1 Molsieve 5A (H_2_ measurement) and Channel 3 CP-SIL 5CB (TOL measurement) were used to measure the gas compositions of the permeate (P1) stream. The gas compositions in the retentate (R1) were calculated from the material balance.

Figure 3b depicts the apparatus diagram for the two-stage gas separation equipment. Here, the pressure of the feed (F1) stream was set at 300 kPaA and controlled using the needle valve. H_2_ and TOL were supplied similarly to the one-stage process (Figure 3a). The permeate (P1) stream from the first stage becomes the feed of the second stage, with a pressure between 111 kPaA and 133 kPaA. The pressure of the feed (F1), permeate (P1), retentate (R1), and permeate (P2) from second-stage streams were measured by a digital pressure manometer (DMS-7A, 200 kPa, Hitachi High-Tech, Tokyo, Japan). The feed H_2_/TOL binary mixture flowed in the direction of the shell side of the membrane module, where the temperature was set at 200 °C. A mass flow meter (Horiba, STEC, Z500 model, Kyoto, Japan) was used to monitor the gas flow rates of the feed (F1), retentate (R1), and permeate (P2) streams. The gas compositions of the permeate (P2) and retentate (R2) streams were determined using Agilent 990 micro gas chromatography, Santa Clara, CA United States. The gas compositions in both permeate (P1) and retentate (R1) were calculated from the material balance.

Partial pressure distributions (concentration distributions) in mixed-gas permeation (one- and two-stage gas separation studies) are produced by selective permeation in the flow direction. Therefore, the driving force for permeation was determined using the logarithmic mean pressure difference for the component of i (∆*p_i_,_lm_*):

(3) ∆pi,lm=∆pi, in−∆pi,outln ∆pi,in∆pi,out

where $∆p_{i,\;in}$ $and\;∆p_{i,out}$ are the partial pressure difference of the *i*-component between the feed and the permeate stream at the inlet and at the outlet, respectively.

The H_2_ purity in the permeate or retentate streams is defined as the ratio of the H_2_ flowrate over total flowrate as follows.

(4) H2 purity in permeate stream=Qp,  H2Qt,p. (100%)

(5) H2 purity in retentate stream=Qr,  H2Qt,r. (100%)

where $Q_{p,H_{2},}$ $Q_{t,p}$, $Q_{r,H_{2}},Q_{t,r}$ are expressed in mol/s, respectively.

The H_2_ recovery in the permeate stream is defined as the amount of H_2_ recovered as the product in the permeate stream, while TOL recovery is defined as the amount of the product recovered in the retentate stream.

(6) H2 recovery in permeate stream=xp,  H2Qt,pxf,  H2Qt,f

(7) TOL recovery in retentate stream=xr, TOLQt,rxf,  TOLQt,f

where the $x_{p,\;H_{2}},\;x_{r,\;TOL},x_{f,\;H_{2}}and\;x_{f,\;TOL}\;$are expressed in mol fraction while $Q_{t,p},\;Q_{t,r}\;and\;Q_{t,f}$ are the flowrates in permeate, retentate, and feed streams, respectively, in L/min unit.

## 3. Results and Discussion

### 3.1. Single-Gas Permeation (SGP) and Pore Size Evaluation

**Figure 4:**
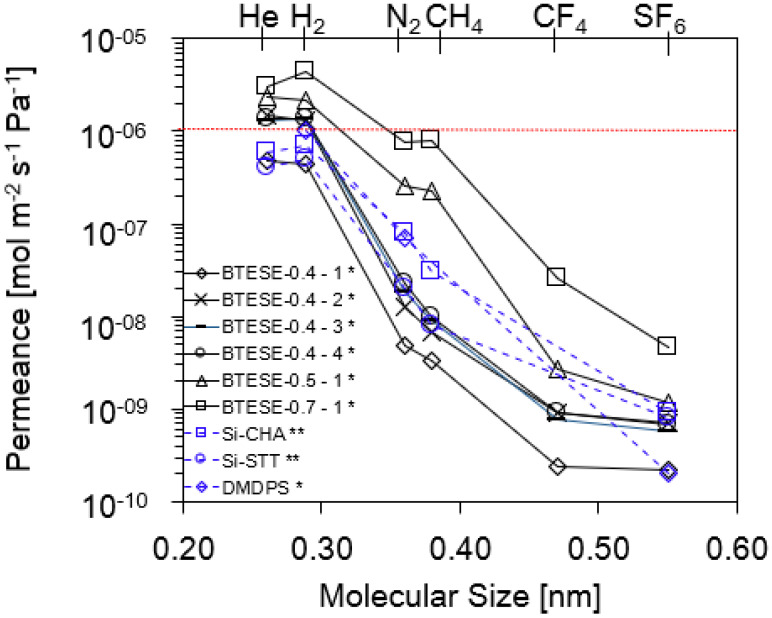
Single gas permeance vs. molecular size (* gas permeation evaluated at 200 °C; ** gas permeation evaluated at 300 °C).

To investigate the permeation effectiveness of the in-house BTESE membranes with other inorganic membranes, the SGP was evaluated using He, H_2_, N_2_, CH_6_, CH_4_, CF_4_, and SF_6_ at a permeation temperature of 200 °C. Si-CHA and Si-STT zeolite membranes [31] evaluated at 300 °C and dimethoxydiphenylsilane (DMDPS)-derived CVD silica membranes [32] evaluated at 200 °C were included, as shown in Figure 4. These two membranes were included in the H_2_/TOL separation study; the authors used an almost similar feed mixture gas (TOL/H_2_ = 2/98 mol%) to our present study (TOL/H_2_ = 3/97 mol%). This condition corresponds to the gas-phase composition after TOL removal by condensation following the methylcyclohexane dehydrogenation process.

Interestingly, despite variations in pore diameters, all of the in-house BTESE membranes showed a H_2_ permeance of more than 10^−6^ mol m^−2^ s^−1^ Pa^−1^, as shown in Figure 4. The BTESE-0.4-1 membrane showed a H_2_ permeance which was lower than other BTESE-0.4 membranes (below 10^−6^ mol m^−2^ s^−1^ Pa^−1^) and is comparable to that of the Si-CHA [31], Si-STT [31], and DMDPS [32] membranes. However, the permeances of other gases such as N_2_, CH_4_, CF_4_, and SF_6_ were higher for both Si-CHA and Si-STT membranes than the BTESE-0.4 type of membrane, demonstrating that both zeolites have a loose pore network compared to BTESE-0.4 membranes. In terms of the DMDPS membrane, it is hard to predict the pore size of this membrane as only a limited number of gases (H_2_, N_2_, and SF_6_) were tested on this membrane, which showed higher N_2_ permeance but similar SF_6_ permeance to the BTESE-0.4-1 membrane.

**Figure 5:**
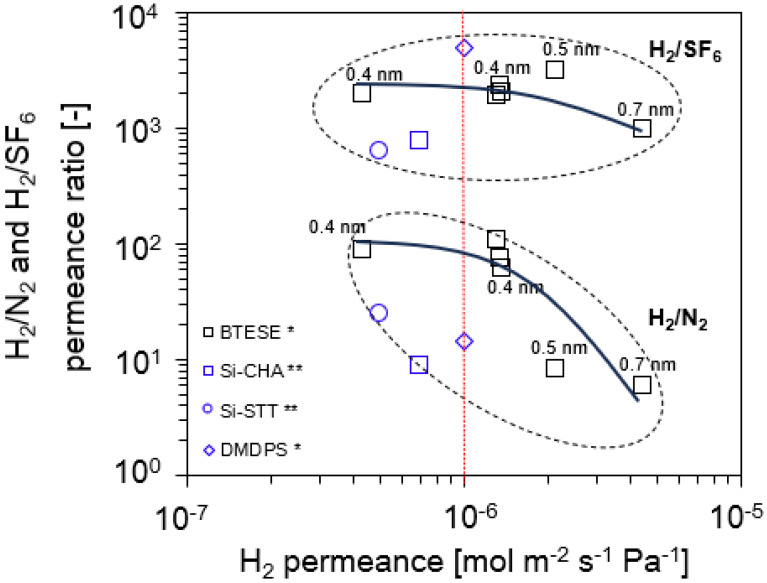
Permeance ratio of H_2_/N_2_ and H_2_/SF_6_ vs. H_2_ permeance (* gas permeation evaluated at 200 °C; ** gas permeation evaluated at 300 °C).

Figure 5 demonstrates the permeance ratios of H_2_/N_2_ and H_2_/SF_6_ as a function of H_2_ permeance. Generally, the performance follows a trade-off relationship for each system (H_2_ vs. SF_6_ and H_2_ vs. N_2_). All BTESE membranes have a moderate H_2_/N_2_ permeance ratio of approximately 5 to 110 but a high and wide range of H_2_/SF_6_ selectivity around 970–2400. The results indicate that when the pore size of the BTESE membranes drops, the gas permeance ratio increases and H_2_ permeance decreases. These results are based on the gas permeation properties and are obtained using the normalized Knudsen-based permeance (NKP) method, which will be discussed later.

In the case of the Si-CHA [31] and Si-STT [31] membranes, the H_2_/N_2_ permeance ratio is 9 and 25, and the H_2_/SF_6_ permeance ratio, on the other hand, is 778 and 625, respectively. Both gas permeance ratios for the Si-CHA and Si-STT membranes are moderate compared to those of the BTESE-0.4 membrane group. This observation supports our previous statement that Si-CHA and Si-STT membranes had a loose pore network compared to the BTESE membrane group. In comparison to the BTESE membranes itself, a H_2_/N_2_ permeance ratio ~ 61 to 106 indicates that the BTESE-0.4 membrane group exhibits a smaller pore size compared to the H_2_/N_2_ permeance ratio < 10 (BTESE-0.5 and BTESE-0.7 membrane groups). The lower H_2_/N_2_ permeance ratio is attributed to the looser structure of the BTESE membrane (BTESE-0.5-1 and BTESE-0.7-1). Large-pore membranes (BTESE-0.5-1 and BTESE-0.7-1) also exhibit high H_2_ permeance due to the low permeation resistance through the membrane. Detailed information on these gas separation properties is summarized in Table 2.

**Figure 6:**
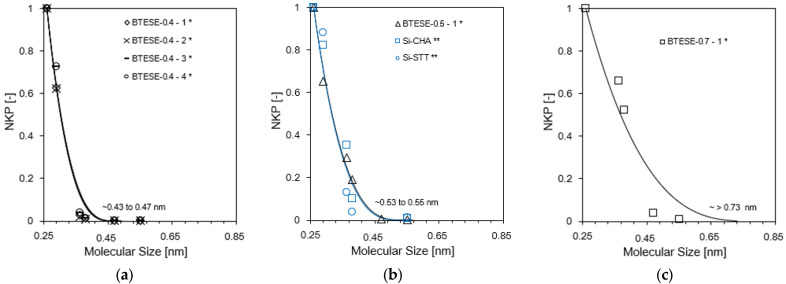
(**a**–**c**) The pore size estimation via the NKP method as a function of the molecular size of permeating gases (symbols and curves indicate the experimental data and the theoretical curves for d_p_ ~0.7, 0.5 and 0.4 nm, respectively). * Gas permeation in this work was evaluated at 200 °C; ** gas permeation in [31] was evaluated at 300 °C.

Figure 6a–c illustrate how the NKP approach [33] was used to precisely assess each membrane’s pore size. The modified gas translation (GT) model, which was developed by Xiao and Wei [34] and Shelekhin et al. [35] for the purpose of determining membrane pore sizes of less than 1 nm, is the foundation of this NKP technique. A more detailed explanation of this model can be found elsewhere [33]. NKP is the ratio of the *i*-th component’s permeance to the permeance predicted by the Knudsen diffusion mechanism using He, the smallest molecule. The normalized Knudsen-based permeance expression can be produced as follows.

(8) $$NKP=\frac{P_{i}}{P_{He}}\frac{\sqrt{M_{i}}}{\sqrt{M_{He}}}=\frac{{\left(d_{p}-d_{k,\;i}\right)}^{3}}{{\left(d_{p}-d_{k,He}\right)}^{3}}exp\left(-\frac{E_{p,\;i}-E_{p,He}}{RT}\right)$$

where *d_p_* is the average pore size of the membrane and $d_{k,\;i}\;$is the molecular size of the *i*-th component. The symbols *E_p,i_*_,_ *E_p,He_*, *R*, and *T* stand for the activation energy of the *i*-th component, He, the gas constant, and the temperature, respectively. To facilitate the evaluation of microporous membrane pore sizes, the current study assumed a small variation in the activation energy of permeation. This led to the following expression for NKP (Equation (9)).

(9) $$NKP=\frac{{\left(d_{p}-d_{k,\;i}\right)}^{3}}{{\left(d_{p}-d_{k,He}\right)}^{3}}$$

Figure 6 shows the NKP plot at 200 °C where the surface flow is negligible. This result confirms that BTESE-0.4 membranes had a smaller pore size distribution.

### 3.2. Hydrogen (H_2_)/Toluene (TOL) Binary Separation in a One-Stage Membrane Configuration System

**Figure 7:**
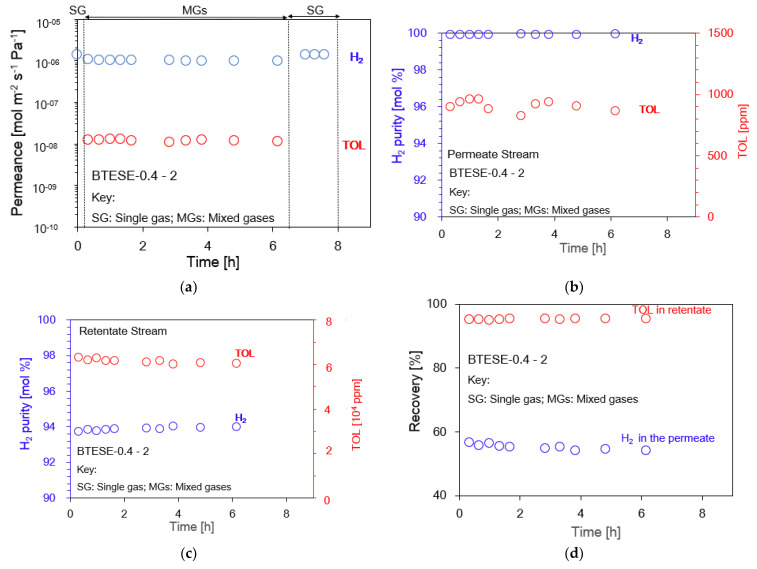
H_2_/TOL separation test for one-stage membrane system configuration, (**a**) H_2_ and TOL permeance; (**b**) H_2_ purity [mol %] and TOL concentration [ppm] in the permeate stream; (**c**) H_2_ purity [mol %] and TOL concentration [ppm] in the retentate stream; and (**d**) H_2_ recovery in permeate and TOL recovery in retentate stream.

The separation performance via a one-stage membrane configuration system is shown in Figure 7a–d. For the 6 h H_2_/TOL separation test, the BTESE-0.4-2 membrane, whose pores are around 0.4 nm in size, is shown as a typical example in the figure. The experiment was initiated with single H_2_, followed by binary separation (H_2_/TOL), and then single H_2_. Compared to the initial single H_2_ gas permeance, the H2 permeance in the mixed H_2_/TOL appears to be slightly lower. However, during the almost 6 h H_2_/TOL separation test, the H_2_ permeance showed stable values > 10^−6^ mol m^−2^ s^−1^ Pa^−1^, with low TOL permeance < 10^−8^ mol m^−2^ s^−1^ Pa^−1^. After the mixed-gas test, H_2_ permeance recovered to the same level as the initial H_2_ single-gas permeance, as shown in Figure 7a. This finding suggests that TOL molecules did not block the membrane pore network or adsorb on its surface. Figure 7b, c shows the permeate H_2_ purity and TOL concentration, which correspond to 99.9 mol% and 900 ppm, respectively. Figure 7d exhibits that the H_2_ recovery in the permeate stream was around 0.5, while the TOL recovery in the retentate stream was 0.9. This study revealed that with only one-stage membranes, the H_2_ purity in the permeate stream can be achieved at approximately ~99 mol% for all BTESE membranes, similarly to other inorganic membranes [31,32,34].

**Figure 8:**
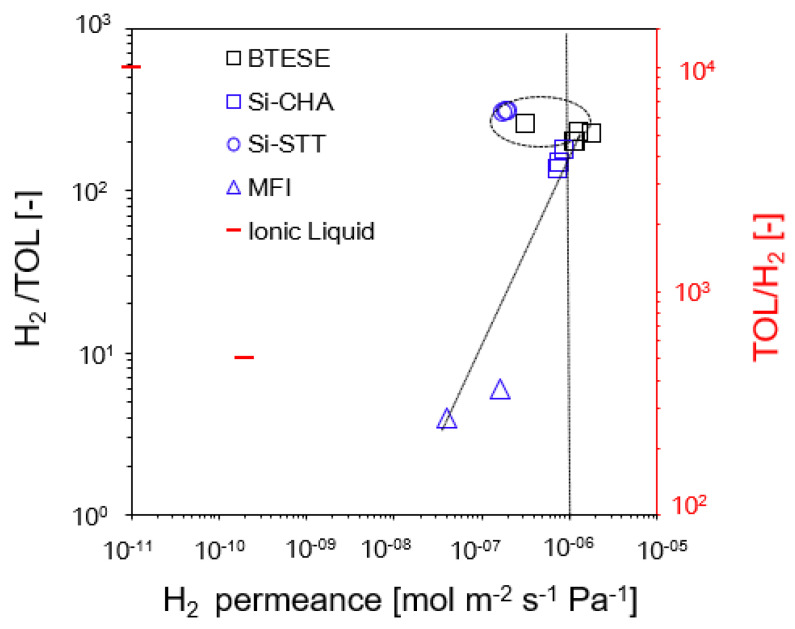
H_2_/TOL and TOL/H_2_ permeance ratios versus H_2_ permeance of BTESE and other inorganic membranes using a one-stage membrane system.

The H_2_/TOL and TOL/H_2_ permeance ratios versus H_2_ permeance of BTESE and other inorganic membranes using a one-stage membrane system are shown in Figure 8. In terms of H_2_ permeance and the H_2_/TOL permeance ratio, our BTESE membranes continue to be regarded as high-performance membranes based on their performance in comparisons with other membrane materials [31,32,36,37]. The ionic liquid membrane [37] is a TOL-permeable membrane as it exhibits a high TOL/H_2_ permeance ratio. However, TOL-permeable membranes are not appropriate for the purification of H_2_.

**Figure 9:**
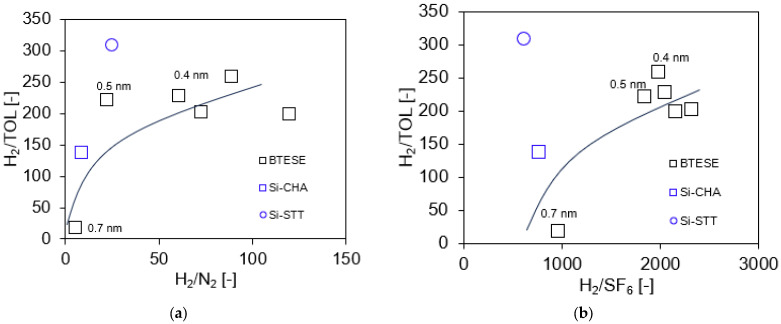
Permeance ratio of the mixed H_2_/TOL versus single-gas H_2_/N_2_ (**a**) and permeance ratio of the mixed H_2_/TOL versus single-gas H_2_/SF_6_ (**b**).

Figure 9 shows the H_2/_TOL permeance ratio plotted against the permeance ratios of H_2_/N_2_ and H_2_/SF_6_ in order to examine any possible correlation. Both the mixed H_2/_TOL and single H_2_/N_2_ and H_2_/SF_6_ permeance ratios were measured at 200 °C. The molecular sizes of hydrogen (H_2_), nitrogen (N_2_), sulfur hexafluoride (SF6), and toluene (TOL) are reportedly 0.29, 0.36, 0.55, and 0.58 nm [14], respectively. Therefore, we assumed it was reasonable to use SF_6_ gas as a predictor for TOL permeance. The permeance ratio can be considered a measurement of the pore size distribution of the BTESE membrane; that is, the higher the permeance ratios, the smaller the pore sizes. As can be seen in Figure 9a,b, with an increase in the permeance ratio of H_2_/N_2_ and H_2_/SF_6_, the permeance ratio of H_2/_TOL also increased. The membranes with small pore sizes (0.4 nm) are plotted at the top right of both figures, as these membranes possessed high separation performance, and the membranes with the membranes with larger pore sizes are plotted at the bottom left of the figures due to their low permeance ratio.

**Figure 10:**
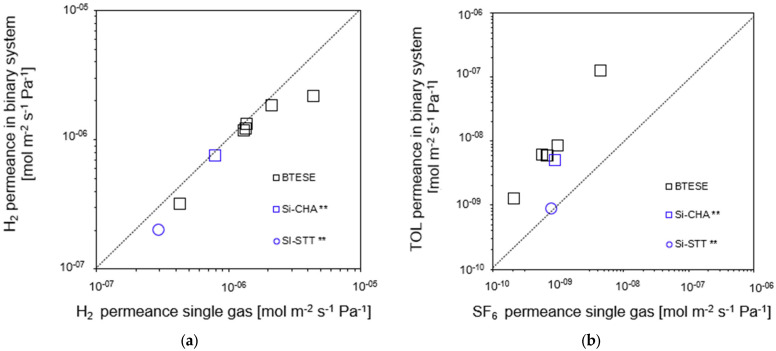
(**a**) H_2_ permeance in binary (H_2_/TOL) and single system and (**b**) TOL permeance versus SF_6_ permeance (* gas permeation evaluated at 200 °C; ** gas permeation evaluated at 150 °C).

Remarkably, Figure 10a shows that the H_2_ mixed-gas permeance was slightly lower than the H_2_ single-gas permeance for all BTESE membranes as well as Si-CHA and Si-STT zeolites membranes. This is possibly due to the fact the fact that some TOL molecules in the mixed-gas system may have absorbed on the membrane surface as well as blocked the pores of the membranes for H_2_ permeation.

However, upon analyzing the TOL and SF_6_ gas permeances, as shown in Figure 10b, we discovered that the TOL permeance was higher than the SF_6_ permeance for all BTESE and Si-CHA membranes. Only the Si-STT membrane shows almost the same permeance as TOL and SF_6_ gas. Toluene (TOL) and sulfur hexafluoride (SF_6_) reportedly have molecular sizes of 0.55 and 0.58 nm, respectively. However, it is possible that TOL’s molecular size was smaller than SF_6_′s. This outcome is consistent with the findings of Niimi et al. [14]. Li et al. [38] reported that the order of permeances was not always correlated with the kinetic diameter. H_2_O and NH_3_ are claimed to have kinetic diameters between 0.26 and 0.265 nm, which is less than the H2 size of 0.289 nm. At high temperatures (400–500 °C), however, amorphous SiO_2_ membranes have consistently demonstrated a higher permeance for H_2_ than for either NH_3_ or H_2_O [38]. The sizes of molecules can be ascertained using a variety of techniques, such as collision diameter and kinetic diameter. According to the findings by Li et al. [38], the appropriate molecular sizes for NH_3_ and H_2_O were found to be 0.326 nm and 0.2995 nm, respectively. An additional possibility is the impact of penetrating molecular structures and membrane pores. Since TOL is most likely more planar than spherical, it may have been preferred for penetration through the slit-like pores or pinholes that may be present in our BTESE-derived membranes. This might be one reason why our BTESE membranes exhibited a greater permeance for TOL in comparison to SF_6_, whose structure is more spherical.

**Figure 11:**
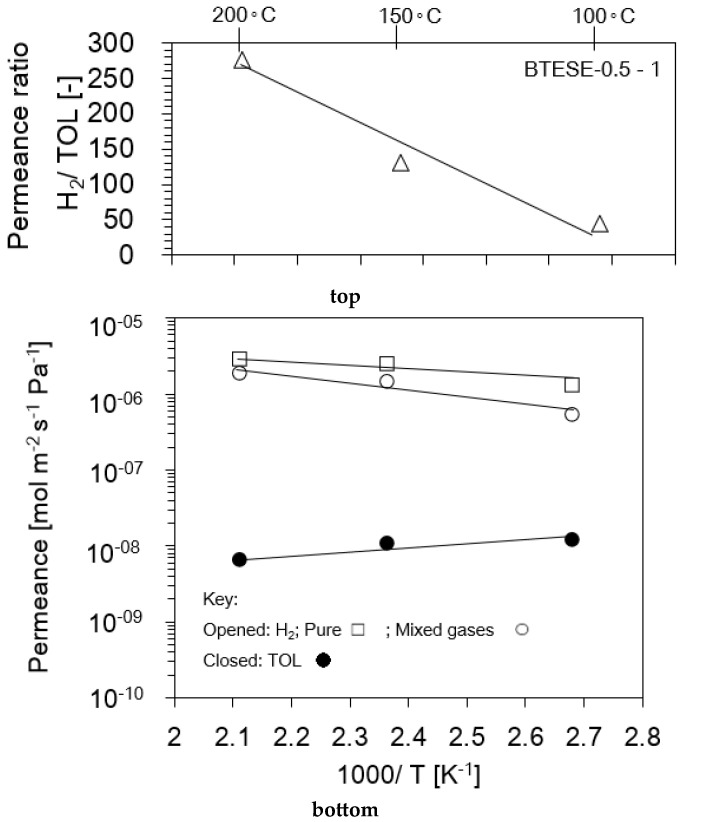
The H_2_ and TOL permeance dependence on temperature (**bottom**) and H_2_/TOL permeance ratio (**top**).

In order to examine the effect of temperature on the BTESE membrane, the BTESE-0.5-1 membrane was tested at different temperatures ranging from 100 to 200 °C, as shown in Figure 10. The feed pressure (*P*_f_) was set at 300 kPaA, and the feed concentrations of H_2_ and TOL were set at 97 mol% and 3 mol%, respectively. Figure 11 illustrates the temperature dependencies of the single H_2_, mixed-gas H_2_, and TOL permeance (bottom figure) and the permeance ratio of H_2_/TOL (top figure), respectively. In spite of the gas state (pure or mixed gas), H_2_ permeance increased as temperature increased, which indicates an activated diffusion tendency [14], whereas TOL permeance decreased as temperature increased in such a manner as to indicate surface diffusion. This result is in line with the finding by Niimi et al. [14] that the effect of TOL adsorption on H_2_ permeation was small at high temperatures. Therefore, it can be concluded that a high-temperature operation is preferable to achieve a high H_2_ permeance and a high H_2_/TOL permeance ratio.

As for the comparison with other membranes, our organosilica BTESE membranes showed high H_2_ permeance compared to the other zeolite membranes [31,32,36]. Most of our BTESE membranes exhibit promising H_2_ permeance over 1 × 10^−6^ mol m^−2^ s^−1^ Pa^−1^ after 60 min of the H_2_/TOL separation test, with the exception of the BTESE-0.4-1 membrane, which has the smallest pore network. BTESE-0.5-1 membrane with a pore size around 0.5 nm exhibits a remarkable performance of H_2_ recovery > 80% and H_2_ purity > 99.9% in the permeate stream. Si-STT membranes showed slightly high H_2_/TOL compared to the BTESE membranes, which was attributed to the adsorption capability of the Si-STT membranes. Reportedly, Si-STT allows toluene to adsorb without preventing H_2_ from passing through its two-dimensional pore systems, which have seven- and nine-membered ring windows (0.24 × 0.35 and 0.37 × 0.53 nm^2^, respectively) [31,37,39,40,41].

Seshimo et al. [32] reported that using a feed mixture of H_2_/TOL (98/2 mol%) at an operating temperature of 200 °C, their dimethoxydiphenylsilane (DMDPS)-derived silica membrane achieved stable purification of hydrogen higher than 99.99% purity and TOL concentrations of 70 ppm in the permeate stream. However, the H_2_ permeance for this membrane was below 1 × 10^−6^ mol m^−2^ s^−1^ Pa^−1^. We also compared our BTESE membrane H_2_/TOL performance with a supported ionic liquid membrane (SILM) and ionic liquid organosilica (ILOS) membranes. These two membranes are reported to have a high TOL/H_2_ separation factor of over 1000–17,000 [37]; however, the H_2_/TOL separation factor is below 1 for these both membranes, and the H_2_ permeance ranges from 1 × 10^−10^ to 1 × 10^−11^ mol m^−2^ s^−1^ Pa^−1^ at operating conditions of feed pressure (*P*_f_: 220 kPaA) and an operating temperature of 70 °C, which seems useful to concentrate TOL but unsuitable to purify H_2_. The detailed information on these H_2_/TOL separation test properties is shown in Table 3.

### 3.3. Hydrogen (H_2_)/Toluene (TOL) Binary Separation in a Two-Stage Membrane Configuration System

Due to the overall performance of our BTESE membrane in terms of TOL concentration in the permeate stream, it still falls short of our desired level, which is less than 200 ppm. In order to reach the intended goal, the two membranes were placed in a cascade (Figure 3b). The BTESE-0.5-1 membrane was chosen as the first membrane, while the BTESE-0.4-2 membrane was chosen as the second membrane for the two-stage membrane configuration system.

**Figure 12:**
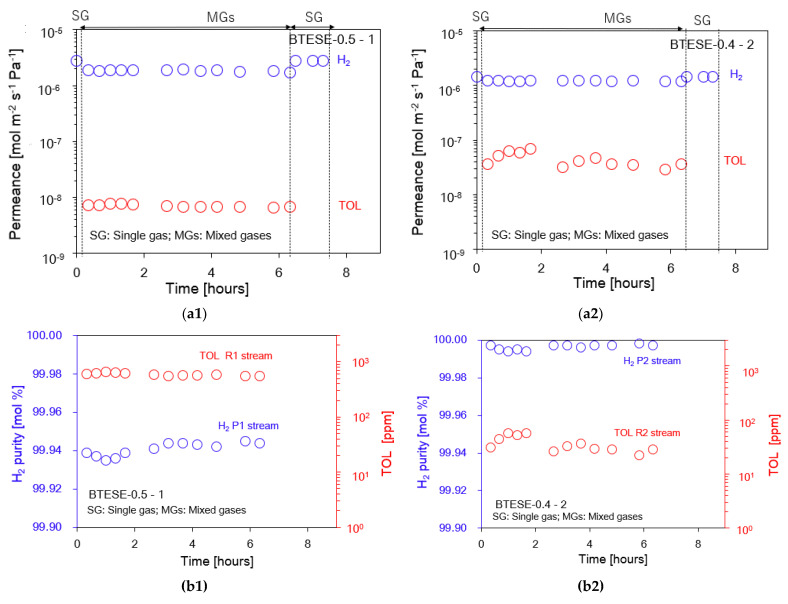

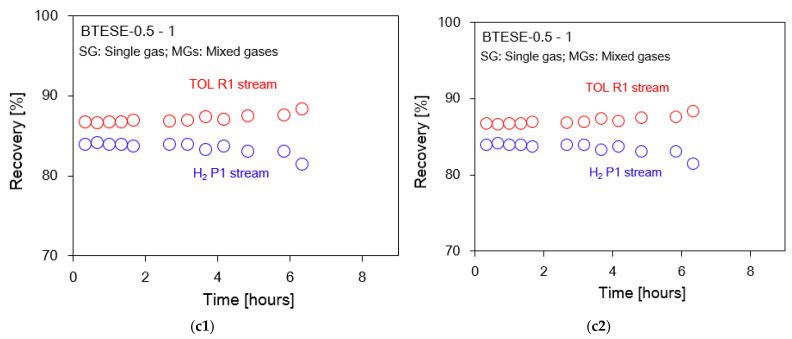
H_2_/TOL separation test for two-stage membrane system configuration. (**a**) H_2_ and TOL permeance in the permeate P1 streams (**a1**) and permeate P2 streams (**a2**); (**b**) H_2_ purity [mol %] and TOL concentration [ppm] in the permeate (P1 and R1) stream (**b1**) and the permeate (P2 and R2) stream (**b2**); (**c**) H_2_ recovery in permeate (P1) and TOL recovery (R1) in retentate streams (**c1**) and permeate (P2) and TOL recovery (R2) in retentate streams (**c2**).

Figure 12 reveals the time course of separation performance of the two-stage membrane configuration system consisting of the BTESE-0.5-1 and BTESE-0.4-2 membranes for an almost 6 h H_2_/TOL separation test. After the 6 h H_2_/TOL separation test, the single H_2_ permeance can still recover to the same value as the initial value of the H_2_ single gas permeance for one- and two-stage membranes, as shown in Figure 12a1,a2. In addition, our target was that the H_2_ permeance should be more than 1 × 10^−6^ mol m^−2^ s^−1^ Pa^−1^. As can be seen after 6 h, the H_2_ permeance for both membranes is still above 1 × 10^−6^ mol m^−2^ s^−1^ Pa^−1^. It can be observed that the H_2_ purity, TOL concentration, and H_2_ and TOL recovery in permeate and retentate for both one- and two-stage membranes were stable for the 6 h H_2_/TOL separation test, as shown in Figure 12b1,b2. The BTESE-0.5-1 membrane enabled H_2_ purity > 99.9 mol% in the permeate (P1) stream; nevertheless, the TOL concentration remained higher than 200 ppm. Due to this reason, we purified the P1 stream with another membrane to reduce the TOL concentration to 200 ppm. By applying the two-stage membrane system, the TOL concentration was reduced to be below 200 ppm, and at the same time, the H_2_ purity was still >99.9 mol% (Figure 12b2). Figure 12c1,c2 show H_2_ recovery in permeate and TOL recovery in retentate in the first and second membranes. More than 80% recovery of H_2_ and TOL was achieved in the first membrane. Approximately 40% H_2_ recovery with a purity of >99.99% was achieved via two-stage configurations.

**Figure 13:**
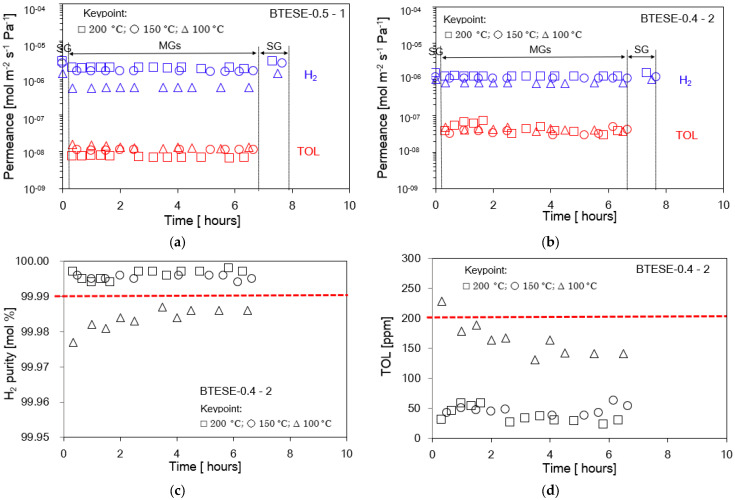
H_2_/TOL separation test at different temperatures (100, 150, 200 °C) for different membrane configurations: (**a**) H_2_ and TOL permeance in the first-stage membrane; (**b**) H_2_ and TOL permeance in the second-stage membrane; (**c**) H_2_ purity [mol %] in the second-stage membrane; and (**d**) TOL concentration in permeate stream in the second-stage membrane.

**Figure 14:**
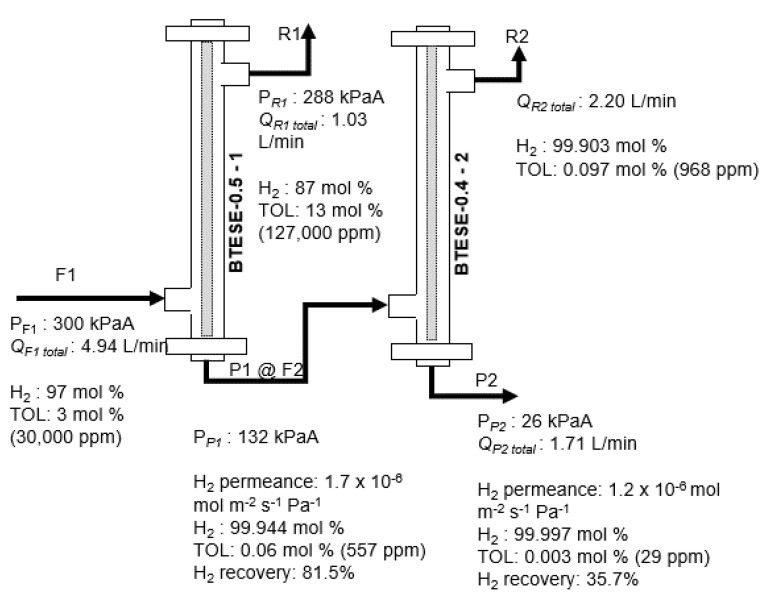
Schematic diagram of the H_2_/TOL separation test for the BTESE-0.5-1 and BTESE-0.4-2 membrane configuration systems at 200 °C.

Meanwhile, Figure 13 shows the time course of the performance of the two-stage membrane configuration system at three different temperatures (100, 150, and 200 °C) for a 7 h H_2_/TOL separation test. It can be seen that at 150 and 200 °C, the H_2_ permeance for both membranes exceeds 1 × 10^−6^ mol m^−2^ s^−1^ Pa^−1^. At temperatures of 150 and 200 °C, the H_2_ purity in the P2 stream is over 99.99 mol%, while the TOL concentration is around 50 ppm. In addition to this, it can be observed that the final product for the H_2_ purity and TOL concentration in the P2 stream as a function of time at temperatures between 150 and 200 °C seems to reach a steady state, probably 2 h after supplying the H_2_/TOL gas mixtures. However, at 100 °C, H_2_ purity and TOL concentration in the P2 stream are not up to the same performance values as at 150 and 200 °C. This is due to the low permeance of H_2_ gas via the one- and two-stage membrane modules, which resulted in a low permeance ratio of H_2_/TOL ~below 50 and 20 (Figure 13a), respectively. Hence, the H_2_/TOL separation test took a longer time to reach its steady state, probably because some TOL molecules gradually blocked the pore network of the membranes; hence, a longer time is required for H_2_ gas to pass through. In addition, there is no additional pressure applied to the two-stage membrane; hence, it can be considered another possible reason for this observation. Based on the final result, 150 and 200 °C are the recommended temperatures for H_2_/TOL separation via the BTESE membrane. Detailed operating data of the two-stage membrane configurations at 200 °C using the BTESE-0.5-1 and BTESE-0.4-2 membrane configuration systems are shown in Figure 14.

## 4. Conclusions

The present study has effectively demonstrated that we can adjust the pore size distribution of our BTESE membranes by changing the number of coating layers. Despite variations in pore diameters, all of the in-house BTESE membranes showed a H_2_ permeance greater than 10^−6^ mol m^−2^ s^−1^ Pa^−1^, with H_2_/SF_6_ > 1000. Additionally, the present study also marks the successful preparation of 40 cm long BTESE membranes for H_2_/TOL separation. The BTESE membranes showed a reasonable correlation for H_2_/TOL against H_2_/N_2_ and H_2_/SF_6_. By applying the two-stage BTESE membrane system configuration, it has been shown that the TOL concentration in the permeate stream can be lowered below 200 ppm while the H_2_ purity is >99.999 mol% and stable for 6 h of operation.

## Figures and Tables

**Table 1:** List of membrane samples.

Membrane Lists	Coating Times of Top Layers	BTESE Sols for Top Layers
BTESE-0.7-1	4	1 wt.% BTESE sol after aging for 8 days at 50 °C
BTESE-0.5-1	5	
BTESE-0.4-1	6	
BTESE-0.4-2		
BTESE-0.4-3		
BTESE-0.4-4		

**Table 2:** Summary of gas permeance, gas permeance ratio, and pore sizes of the BTESE and other inorganic membranes.

Membrane Numbers	H_2_ Permeance [10^−6^ mol m^−2^ s^−1^ Pa^−1^]	Permeance Ratio		NKP [nm]
		H_2_/N_2_	H_2_/SF_6_	
BTESE-0.4-1 *	0.43	89	1991	~0.46
BTESE-0.4-2 *	1.33	106	1908	~0.46
BTESE-0.4-3 *	1.36	73	2321	~0.47
BTESE-0.4-4 *	1.38	61	2051	~0.47
BTESE-0.5-1 *	2.15	8	1846	~0.54
BTESE-0.7-1 *	4.45	6	972	~0.73
Si-CHA [31] **	0.70	9	778	~0.55
Si-STT [31] **	0.50	25	625	~0.53
DMDPS [32] *	1.00	14	5000	N/A

* Gas permeation evaluated at 200 °C; ** gas permeation evaluated at 300 °C.

**Table 3:** H_2_/TOL separation test performance of BTESE and other inorganic membranes via one-stage membrane process.

Membrane Code	Pressure [kPaA]		Temperature [°C]	H_2_/TOL Concentration Feed mol [%]	Permeate Stream						Retentate Stream		Refs
					Permeance [10^−6^ mol m^−2^ s^−1^ Pa^−1^]		H_2_/ TOL [-]	H_2_ Purity [mol %]	H_2_ Recovery [%]	TOL Concentration [ppm]	TOL Recovery [%]	TOL Concentration [ppm]	
	Feed	Permeate			H_2_	TOL							
BTESE-0.4-1	200	101.3	200	97/3	0.32	0.00123	258	99.972	8.8	280	98.2	32,668	[This work]
BTESE-0.4-2	200	101.3	200	97/3	1.16	0.00586	198	99.957	30.2	428	98.5	41,730	[This work]
BTESE-0.4-2	300	111.0	200	97/3	0.96	0.01104	87	99.914	53.9	859	95.3	60,149	[This work]
BTESE-0.4-3	200	101.3	200	97/3	1.21	0.00602	200	99.958	34.1	418	95.9	43,977	[This work]
BTESE-0.4-4	200	101.3	200	97/3	1.31	0.00579	227	99.963	38.7	375	100.1	46,972	[This work]
BTESE-0.5-1	200	101.3	200	97/3	1.83	0.00830	221	99.956	50.2	441	96.1	56,490	[This work]
BTESE-0.5-1	300	101.3	200	97/3	1.73	0.00681	253	99.944	81.5	557	88.0	127,303	[This work]
BTESE-0.7-1	200	101.3	200	97/3	2.15	0.12300	17	99.456	60.0	5441	92.0	62,293	[This work]
Si-CHA	300	101.3	90	98/2	0.88	0.00500	176	99.989	N/A	N/A	N/A	N/A	[31]
Si-CHA	300	101.3	120	98/2	0.77	0.00520	148	99.987	N/A	N/A	N/A	N/A	[31]
Si-CHA	300	101.3	150	98/2	0.75	0.00550	136	99.984	N/A	N/A	N/A	N/A	[31]
Si-STT	300	101.3	90	98/2	0.18	0.00060	300	99.992	N/A	N/A	N/A	N/A	[31]
Si-STT	300	101.3	120	98/2	0.19	0.00062	306	99.992	N/A	N/A	N/A	N/A	[31]
Si-STT	300	101.3	150	98/2	0.20	0.00065	308	99.991	N/A	N/A	N/A	N/A	[31]
DMDPS	N/A	N/A	200	98/2	0.90	N/A	N/A	99.996					[32]
MFI	300	101.3	120	98/2	0.04	0.00940	4	N/A	N/A	N/A	N/A	N/A	[36]
MFI	300	101.3	200	98/2	0.16	0.02500	6	N/A	N/A	N/A	N/A	N/A	[36]
SILM	220	200	70	N/A	0.0002	0.10	0.0020	N/A	N/A	N/A	N/A	N/A	[37]
ILOS	220	200	70	N/A	0.00001	0.10	0.0001	N/A	N/A	N/A	N/A	N/A	[37]

## Data Availability

The raw data supporting the conclusions of this article will be made available by the authors on request.
